# Outlier Responses Reflect Sensitivity to Statistical Structure in the Human Brain

**DOI:** 10.1371/journal.pcbi.1002999

**Published:** 2013-03-28

**Authors:** Marta I. Garrido, Maneesh Sahani, Raymond J. Dolan

**Affiliations:** 1 University College London, Wellcome Trust Centre for Neuroimaging, London, United Kingdom; 2 The University of Queensland, Queensland Brain Institute, St Lucia, Queensland, Brisbane, Australia; 3 University College London, Gatsby Computational Neuroscience Unit, London, United Kingdom; Indiana University, United States of America

## Abstract

We constantly look for patterns in the environment that allow us to learn its key regularities. These regularities are fundamental in enabling us to make predictions about what is likely to happen next. The physiological study of regularity extraction has focused primarily on repetitive sequence-based rules within the sensory environment, or on stimulus-outcome associations in the context of reward-based decision-making. Here we ask whether we implicitly encode non-sequential stochastic regularities, and detect violations therein. We addressed this question using a novel experimental design and both behavioural and magnetoencephalographic (MEG) metrics associated with responses to pure-tone sounds with frequencies sampled from a Gaussian distribution. We observed that sounds in the tail of the distribution evoked a larger response than those that fell at the centre. This response resembled the mismatch negativity (MMN) evoked by surprising or unlikely events in traditional oddball paradigms. Crucially, responses to physically identical outliers were greater when the distribution was narrower. These results show that humans implicitly keep track of the uncertainty induced by apparently random distributions of sensory events. Source reconstruction suggested that the statistical-context-sensitive responses arose in a temporo-parietal network, areas that have been associated with attention orientation to unexpected events. Our results demonstrate a very early neurophysiological marker of the brain's ability to implicitly encode complex statistical structure in the environment. We suggest that this sensitivity provides a computational basis for our ability to make perceptual inferences in noisy environments and to make decisions in an uncertain world.

## Author Summary

Survival crucially depends on our ability to extract information from the environment. This ability relies on learning about regularities that enable us to make predictions about what is likely to happen next. Sensitivity to violations of these regularities is necessary for timely reactions and adaptive responses to unexpected, or odd, events. Prior work on speech acquisition and artificial grammar learning has provided important behavioural evidence that humans are able to learn statistical regularities, but it still falls considerably short of providing a biological understanding for how these processes might take place in the brain. The neurophysiological study of regularity extraction has so far been limited, to either sequence-based rules or to simple change-detection paradigms, and thus the neurobiological mechanisms that underpin statistical learning remain unknown. Here we provide both behavioural and neurophysiological evidence to show that humans keep track of the uncertainty in apparently random distributions of events. Our work demonstrates that an early neurophysiological signal underlies the fundamental human ability of learning and making inferences in an uncertain world.

## Introduction

The survival of an organism often depends on its ability to form expectations about the structure of its sensory environment, and to monitor the environment for violations of these expectations so as to respond to unexpected and potentially threatening events [1]–[5]. In many instances this goal is rendered challenging by the unpredictability of even the normal environment [6].

Several studies have examined an ability that humans have to implicitly learn regularities in observed stimuli [7]. In the auditory domain many of these studies have used an oddball paradigm [8], in which participants are presented with a sequence of events that mostly obey a certain rule, but which is punctuated by occasional “oddballs” or events that violate that rule. These oddballs frequently evoke conspicuous neurophysiological activity, reflected in the so-called mismatch negativity (MMN) response. The MMN can be observed in electro- and magneto-encephalographic recordings (EEG and MEG) with a time latency of about 100 to 200 ms from violation onset [8]. In the classical, and simplest, oddball paradigm, the main sequence comprises identical tonal stimuli called “standards”. The oddballs are events that differ from the standards in some physical aspect such as frequency [9]–[12], duration [13], or amplitude [14]. The MMN response is robustly elicited in all these cases and represents a neurophysiological marker both of the internalisation of the regularity, and of the change detection.

Other experiments have observed MMN signals associated with the violation of more sophisticated rules. Examples include: a tonal sequence in which the higher the frequency of a tone, the louder its amplitude, with violation by a high-frequency soft or low-frequency loud tone [15]; a sequence of regularly descending tone pairs broken by an occasional ascending combination [16]; or a regular rhythmic pattern violated by an unexpectedly-timed event [17].

Despite the sophistication of these regularities, and the occasional randomisation of any aspects of the standard stimuli that are irrelevant to the rule [13], these studies predominantly rely on establishing a deterministic, often sequence-based pattern [18], [19], against which oddballs may be judged. A few studies have introduced some variability into the distribution of standards and observed that MMN amplitude decreased when the range of variability increased [20]–[22]. However, in these studies the “oddball” was still outside the distribution of standards, and the standards were chosen from a small set of discrete known tones, to which listeners had become accustomed. By contrast, we were interested in whether human listeners implicitly learn a *statistical* regularity in a stream of unknown, continuously-distributed, *stochastic* stimuli: a simple probabilistic pattern that could not be encoded as a deterministic sequence-based rule, a finite set of known standard stimuli, or by a categorical separation between expected events and outliers. In our study, the definition of the standards, or the context, is entirely probabilistic. As opposed to situations [20], [22] where oddballs were clearly outside the range of standard variability, here both standards and oddballs are part of the same distribution. Importantly, by its nature, such regularity does not provide for a categorical separation between expected events and outliers. Instead, as expectations are themselves uncertain, oddball events must be defined quantitatively in terms of how unlikely, or surprising they are given the probabilistic expectations formed by the majority of stimuli. Thus, our prediction was that a comparison between the neurophysiological responses evoked by very likely events to those evoked by very unlikely events should reveal an MMN-like deflection. Moreover, the strength of this MMN should depend on the relative likelihoods of the stimuli being compared. In particular, by establishing two different statistical contexts, with two different distributions of expected events, we could manipulate the probability associated with the occurrence of the very same physical stimulus in each of the two contexts.

Our key prediction here was that the stimulus should evoke a larger prediction error, or MMN response, when embedded within the context in which it was less likely. In effect, by investigating human listeners' sensitivity to statistical outliers embedded within a distribution of random stimuli, we could determine both whether listeners are able to implicitly learn and encode a statistical pattern, and whether detection of outliers from this pattern evokes an MMN signal similar to that evoked by violations of deterministic sequences.

## Results

### Experiment 1: Behavioural evidence for statistical sensitivity

**Figure 1:**
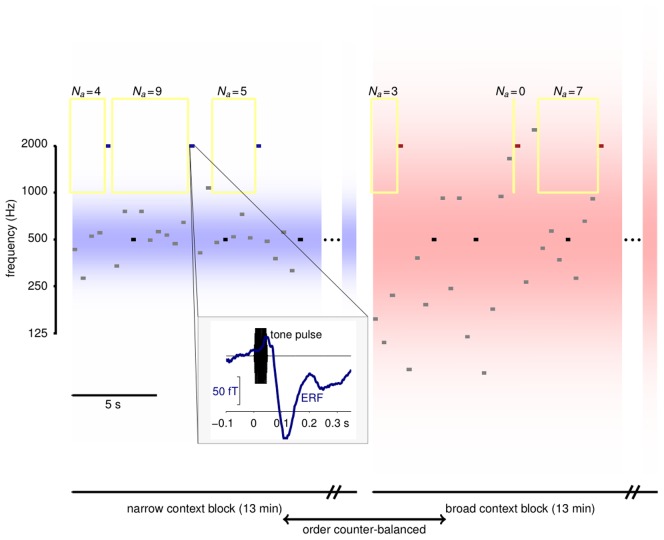
Experimental design. Tone pulse sequences were presented in blocks. The frequencies of the majority of tones in each block (*grey*) were drawn from a contextual distribution that could be narrow (left) or broad (right). The distribution densities are shown in blue and red shading; both were centred at 500 Hz and had standard deviations of 0.5 and 1.5 octaves respectively. Embedded in both sequences were probe tones whose frequencies were either equal to the distribution centres (standard, *black*), or 2 octaves above (odd, *blue* and *red*). *Yellow* rectangles indicate frequency exclusion windows used for the local adaptation analysis of Figure 5; the associated values of 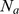 give the number of preceding tones that fell outside the adaptation window. Block lengths are indicated for Experiment 2. Blocks in Experiment 1 were shorter and repeated more often. Inset: The timing of tones and MEG epochs. Tone pulse waveforms (*black*) lasted 50 ms with ramped onsets and offsets. MEG responses (*blue*) were extracted from 100 ms before to 350 ms after tone onset. The evoked response shown is the average response to odd probes in the narrow context, spatially filtered as in Figure 5.

Ten human participants reported changes in the luminance of a fixation cross while ignoring a background sound made up of tone pulses. The frequencies of most of the tone pulses in the background sounds were drawn from either a narrow or a broad distribution both of which were Gaussian in the log domain (Figure 1). These distributions provided two different contexts for the presentation of probe tones that were interspersed within the random streams. The probe tone frequencies were either equal to the centre frequency of the contextual distributions (500 Hz, likely or “standard”), or two octaves above it (2000 Hz, unlikely or “odd”). In fact, in this first experiment each probe tone coincided with a change in luminance in the fixation target, but participants were not informed of this.

**Figure 2:**
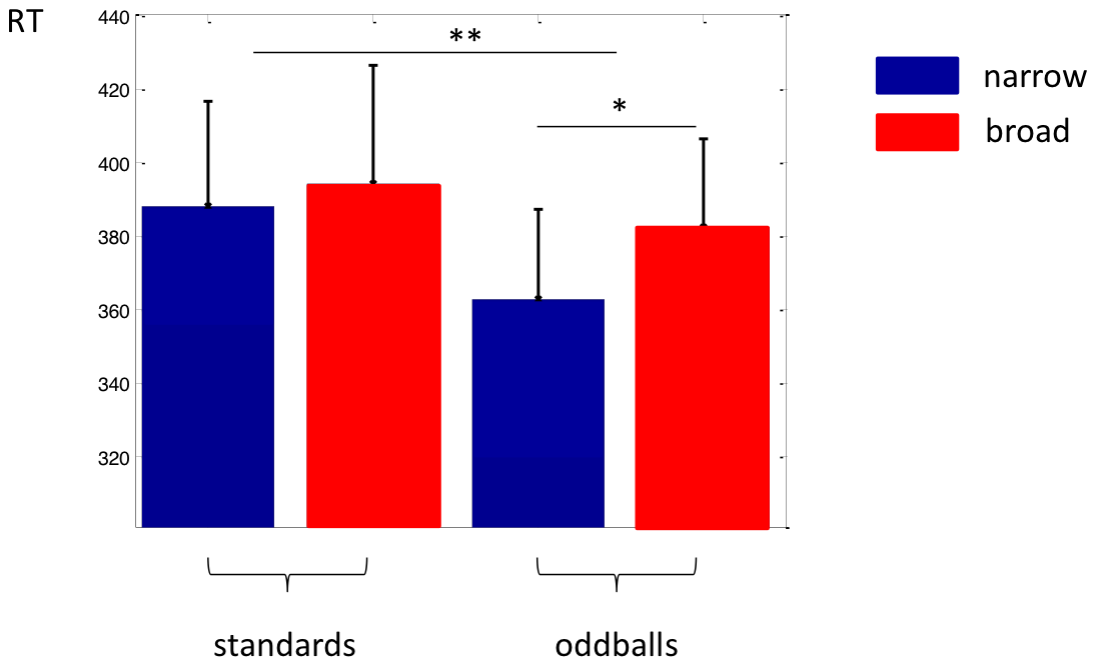
Behavioural results. Displayed are responses to standard and odd probe tones under the narrow (in *blue*) and broad (in *red*) contexts. Reaction time was significantly shorter when it was accompanied by an odd probe tone rather than a standard tone regardless of the context (p = 0.002, ANOVA main effect), and more so when luminance changes were paired with odd probe tones in the narrow compared to the broad context (p = 0.043, ANOVA interaction, p = 0.0076 post-hoc t-test).

Nonetheless, we found that the reaction time to the luminance change was shorter when it was accompanied by an odd probe tone rather than a standard tone regardless of the context (p = 0.002, ANOVA main effect, see Figure 2). This finding is consistent with earlier observations that auditory outliers embedded in simple deterministic patterns also facilitate visual target detection [23]. We also found that reaction times were shorter overall in the narrow as compared to the broad context (p = 0.004, ANOVA main effect) and, crucially, that responses to luminance changes paired with odd probe tones in the narrow context were faster than those to changes paired with the same odd probe tones in the broad context (p = 0.043, ANOVA interaction, p = 0.0076 post-hoc t-test).

Thus, these behavioural data show that listeners were indeed sensitive to the contextual distribution and its associated probabilities. Although the odd probes themselves were embedded at the same rate in both contexts, when tones of similar frequency were less likely to occur by chance within the background distribution, then the associated facilitation of behavioural responses was stronger.

### Experiment 2: Neurophysiological evidence for statistical regularity encoding

We next asked whether sensitivity to contextual statistical properties had a neurophysiological fingerprint. To avoid possibly confounding motor or attentional signals associated with the visual task, we modified the experimental design to break the association between probe sounds and visual events. Thus, in this variant of the task, the visual and auditory streams were entirely unrelated. Eighteen naïve participants performed this modified task while we recorded neural activity with MEG. As before, participants were presented with probe sounds embedded in two contextual frequency distributions characterised by equal means and different variances, so that physically-identical odd probes were more unlikely under the narrow (low variance) than under the broad (high variance) distribution. These two distributions were presented in two separate blocks and the block order was counter-balanced across participants to avoid order effects (Figure 1).

### Scalp analysis

**Figure 3:**
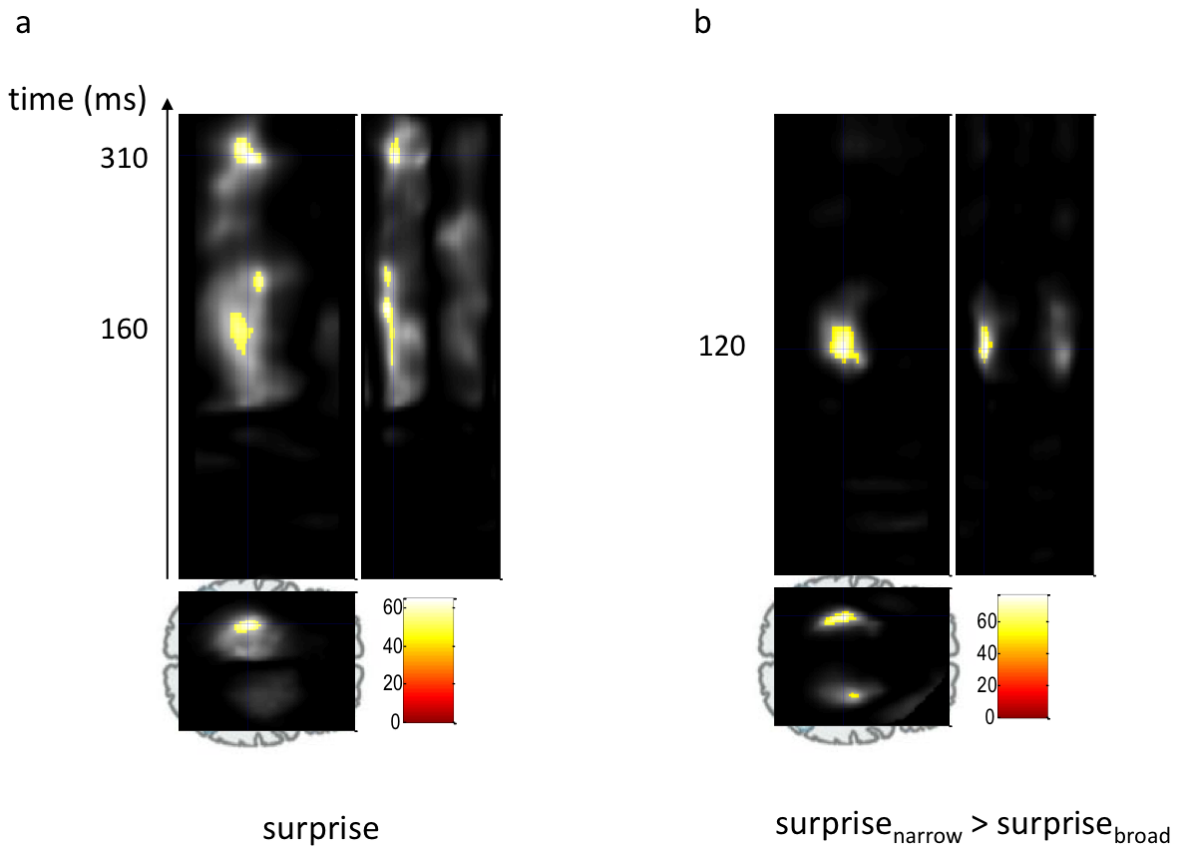
Sensitivity to statistical contexts in brain scalp data. Spatio-temporal statistical analysis reveals significant effects over bilateral temporo-parietal areas (displayed at p<0.05, FWE whole-volume corrected) for (**a**) the main effect of surprise - deviance from the mean (odd vs. standard, MMN-like response) peaking at about 160, 190, and 310 ms and (**b**) the interaction, i.e., differences between MMNs under the low and high variance contexts, peaking at about 120 ms.

We performed a full spatio-temporal statistical analysis, searching for significant differences between the magnetic fields evoked by odd and standard probe sounds (evoked response fields or ERFs), treating these probes as analogous to the oddballs and standard events respectively in a classical MMN paradigm. At the scalp level, we found three left-lateralised clusters (family wise error (FWE) corrected at p<0.05) peaking at about 160, 190, and 310 ms over temporal-parietal areas (Figure 3a). These corresponded in latency and shape to the traditional MMN and P3a components, as predicted. Thus, this finding suggests that in a background of tones with randomly-distributed frequencies, sounds whose frequencies lie distant from the mean are registered physiologically as outliers and treated differently to tones whose frequencies fall at the centres of the distributions. However, although the timing, form and localisation of this response are all reminiscent of such a statistically-driven MMN effect, we could not rule out a contribution due to the differing tone frequencies of the odd and standard probes.

Thus, the crucial test was our second prediction: that the size of the outlier response (odd probe response minus standard probe response) would be greater in the context of the narrow (relative to the broad) distribution. This is exactly what we found: an MMN-like response peaking at about 120 ms over bilateral temporal-parietal areas, which was stronger (more negative) in the context of the narrow distribution (Figure 3b). Here, the comparison is between responses evoked by physically identical sounds; the only difference lies in the context, with the frequency of the odd probe tone being much less likely to occur under the narrow distribution. This difference in the magnitude of the MMN-like response was mediated by context-dependent changes in the amplitude of both the responses to odd and to standard probes; responses to standard probes were smaller and those to odd probes were larger when the same two sounds were played in the narrow context (Figure S2).

### Source analysis

We then reconstructed putative magnetic-field sources from the scalp activity (using a multiple sparse priors MSP inverse solution [24], [25]), in order to infer the cortical regions most likely to have generated the signals observed in the expected MMN time-window (between 100–200 ms from stimulus onset). As described above, the differences in fields measured at the scalp were clearly statistically significant, even when we accounted for the fact that tests were repeated for each electrode and each time point. Here, we sought to identify the most likely sources of these significant effects. As the number of cortical voxels considered was much larger than the number of electrodes, differences in reconstructed source activity generally did not appear significant after naïve correction for multiple testing – even though the significant scalp-field differences must, of course, originate from sources somewhere in the brain. Thus, we identified putative source locations using uncorrected (p<0.05) significance thresholds, and then performed a set-wise significance analysis using anatomical masks defined by prior studies. This procedure does leave open the possibility of errors in the precise localisation of activity within each defined set.

**Figure 4:**
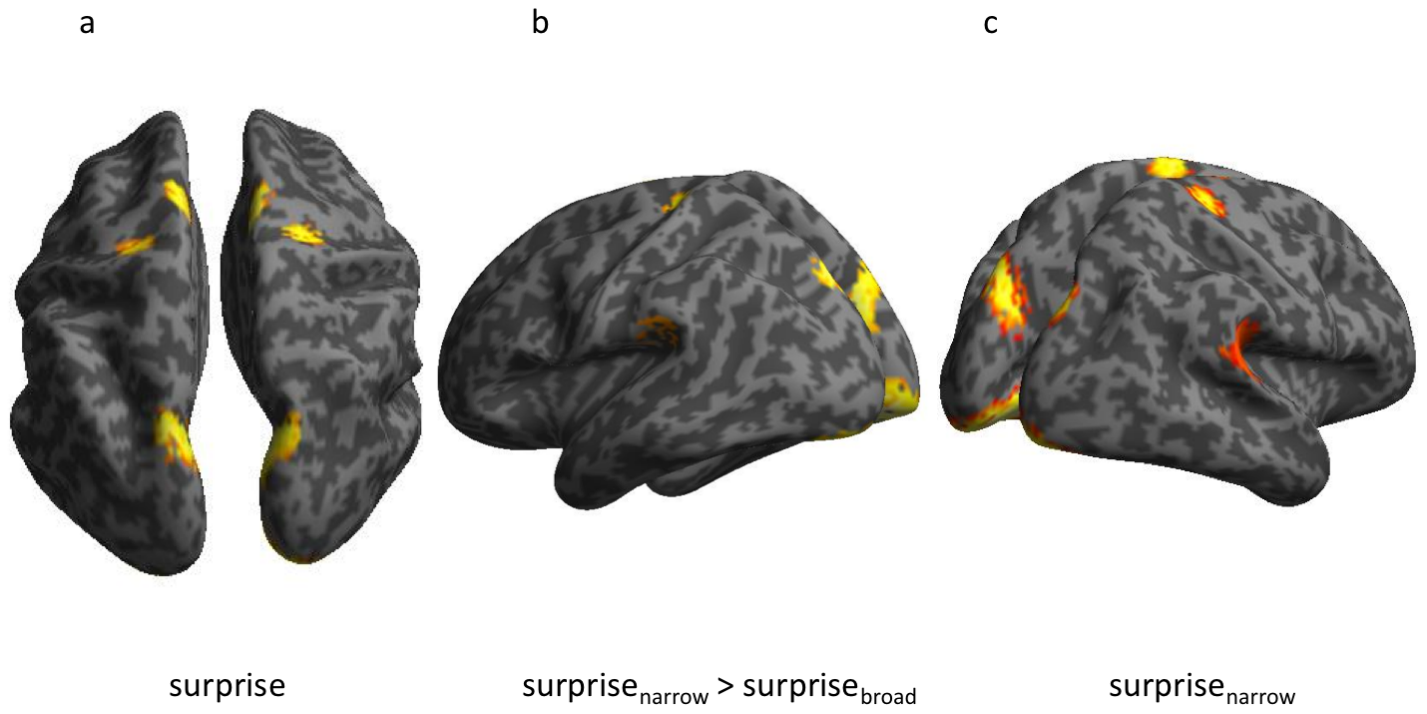
Sensitivity to statistical contexts revealed at cortical sources. Source reconstruction analysis reveals: (**a**) main effect of surprise (larger sensitivity to odd vs. standard probes), (**b**) larger MMN-like effects under the narrow than the broad distribution and (**c**) a simple main effect of surprise. (All effects are displayed at p<0.05, uncorrected).

We found a main effect of surprise in bilateral visual, parietal, and sensory-motor cortices (p<0.05, uncorrected) (see Figure 4a). We also found an interaction effect (p<0.05, uncorrected) in similar areas as well as in auditory association cortex, manifest in a stronger response to odd probes under the narrow compared to the broad distribution. Although these effects were not significant when corrected for the multiple comparisons performed over the whole brain, they were included in sets that showed significant effects (p<0.05, corrected) when defined by anatomical masks derived from previous independent studies (WFU Pick Atlas [26]) for regions expected *a priori*, such as the effects seen in temporal [9], [27]–[30] and the parietal [28], [30] regions. Thus, these sources showed MMN-like responses that were larger when the probes were more unlikely under the contextual distribution (Figure 4b).

As opposed to what we had predicted, we did not find a main effect of surprise in the associative auditory cortex [9], [27], [29]. In the narrow context alone, there was indeed a simple main effect of surprise in the associative auditory cortex (Figure 4c). However, the same contrast did not reveal an effect under the broad distribution (even at p<0.1). This might indicate that an MMN-like response is generated in the auditory cortex only in the narrow context, where the odd probes are more unlikely, although it may also just reflect an interaction between a larger MMN effect-size in the narrow context and a noisier reconstruction of auditory cortical sources than parietal ones.

### Local adaptation

The analyses so far show that there is an MMN-like differential response to sounds that are unlikely in a statistical context, when contrasted with higher probability sounds, and that this difference is stronger when the difference in probabilities is greater. There has been some debate about the extent to which MMN-like responses to frequency deviants may be mediated by frequency- and temporally-local adaptive processes [31]–[34]. A number of different mechanistic theories for MMN generation have been discussed in great depth (see [35], [36] for reviews). One explanation rests on the fact that changes lead to release from adaptation to repeated events, or refractoriness, resulting in an enhanced response to a novel stimulus [33], [34]. While this theory is useful in the case of repeated standards, it does not explain very well why MMN is elicited by more abstract rules that do not involve change, or a break in repetition [15], [16]. Recent efforts to disentangle refractoriness and memory-comparison-based contributions to MMN have been able to demonstrate that there is more to MMN than simple adaptation [37]. However, in light of this debate, it was important to determine how far local adaptive or refractory processes might have contributed to the phenomena we observed.

Although tones with exactly the odd probe frequency never occurred by chance under either contextual distribution, tones with nearby frequencies did, and did so more often in the broad context than in the narrow. Thus a local spread of adaptive effects, whereby these contextual tones reduce the size of physiological response to tones of nearby frequencies including the odd probe tones, might contribute to the difference in response magnitudes between the two contexts. On a long-time scale spanning multiple stimuli [38], such an effect could mediate the physiological mechanism underlying sensitivity to environmental statistics. However, if the bulk of the effect were due to adaptation arising from very recent stimulation, then there would be less reason to posit a mechanism that accrues and responds to longer-term regularities.

To study the contribution of local adaptation, we grouped responses to odd probe sounds according to the number of preceding sounds, 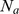, that fell *outside* a frequency window of width 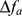 specified in octaves and centred on the frequency of the odd probes (Figure 1). Responses to tones with larger 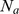 would be expected to suffer less short-range adaptation. We used window widths ranging from one-third of an octave (roughly the equivalent rectangular bandwidth, ERB, of a psychoacoustically-defined ‘auditory filter’ [39]) to five times the ERB. Wider windows yielded too few data to be interpretable.

**Figure 5:**
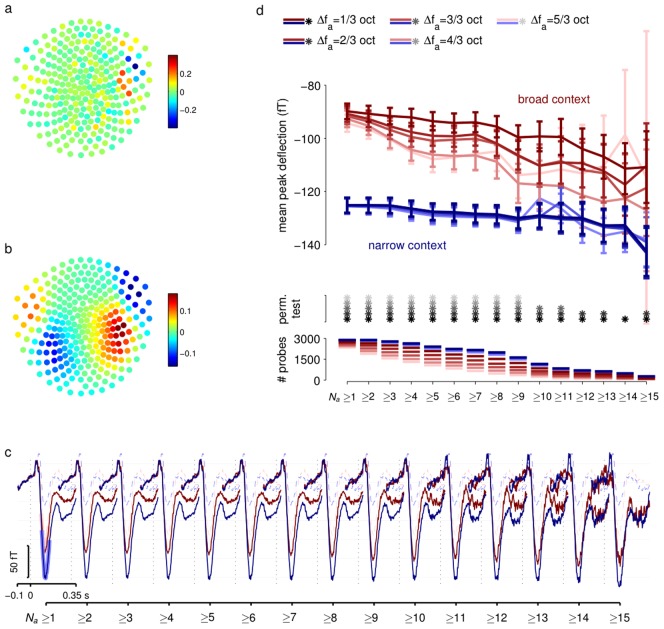
Local adaptation effects. (**a**) Pattern of weights of the spatial filter used to extract the maximal signal-to-noise spatial projection of the MEG data. (**b**) The implied spatial pattern of the signal extracted by the filter shown in (**a**). (**c**) ERFs obtained by averaging responses to odd probes (*solid lines*) selected by threshold value of *N_a_* (number of preceding tones falling outside a frequency window of width 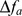, here 1/3 octave). ERFs are separated by context (*blue* narrow; *red* broad). Shading for 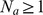 curves show regions averaged to obtain peak values in (**d**). Adaptation is evident for odd probes in the broad context but small or absent in the narrow context. ERFs for standard probes (*dashed lines*) are also shown for reference, and are not grouped by *N_a_*. (**d**) Adaptation effects for a range of windows. Curves show ERF peaks (averaged as indicated in (**c**)) for odd probes in narrow (*blue*) and broad (*red*) context as a function of threshold value of *N_a_*, calculated for different frequency exclusion windows (*colour saturation*, see legend at top). Error bars show standard errors. Grey stars indicate pairs of ERFs that were significantly different at the p<0.05 level according to a random permutation test. Lines at the bottom show the number of probe tones (combined across all subjects) that contribute to each ERF. Numbers fall as threshold *N_a_* grows, contributing to greater uncertainty in measurements.

As there were relatively few responses contributing to each average ERF in this analysis, particularly for larger values of 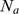, we applied a spatial projection filter to find a single weighted combination of all the MEG channels, common to all participants, that maximised the signal-to-noise ratio of the filtered signal for all four types of probe sounds [40]: standard and odd in narrow and broad contexts (Figure 5a, b). Importantly this spatial filter was not designed to accentuate differences between the responses to the different probes. Indeed, depending on the geometry of the signals and noise, it might be expected to slightly suppress the magnitude of these differences, whilst simultaneously reducing noise in each signal.

Figure 5c shows the filtered components associated with odd probe sounds selected according to increasing minimum values of 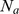, using the ERB-sized window, as well as the filtered components for standard probes without selection by 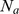, for both distributional contexts. As the threshold value of 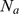 increases, the peak amplitude of the response to odd probes in the broad context grows more negative, presumably reflecting a contribution from local adaptive mechanisms. However, even when the preceding 15 tones (lasting 7.5 s) fell outside the ERB window, the response to odd probes in the broad context does not reach the same level as that to odd probes in the narrow context (p<0.05 random permutation tests applied separately for each threshold). This general result held true for a range of exclusion windows 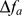 (Figure 5d), with the mean ERFs remaining systematically separate, although for the larger exclusion windows there were too few stimuli with large enough values of 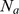 for the effect to still reach significance. The same observation held when responses were grouped according to small ranges of 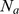, rather than by threshold values (Figure S1). Thus, the difference in response magnitude to odd probe sounds in the two distributional contexts cannot be attributed solely to local adaptation mechanisms, and depends on the distribution of stimuli well outside the auditory filter or more than 15 stimuli or 7.5 s in the past.

A striking and unexpected observation was that the response to odd probes in the narrow context seemed to be relatively unaffected by 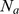. This might well reflect a difference in the adaptive impact of other odd probe sounds when compared to tones whose frequencies are drawn from the distributional context. In the narrow context most preceding tones that fell within 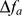 were themselves odd probe sounds. The fact that very little adaptation is seen even when such a tone happens to have fallen in the very recent past (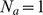 or 2, see Figure S1), raises the intriguing possibility that once isolated tones are marked by a physiological mechanism as outliers, they have only reduced, or even non-existent, adaptive impact on subsequent tones.

## Discussion

We provide behavioural and neurophysiological evidence that humans implicitly track statistical regularities of the sensory environment. Specifically, our findings show that stimuli that fall outside an established stochastic pattern evoke behavioural and neurophysiological responses previously associated with violations of a repetitive or deterministic sequence. Furthermore, we found that exactly the same physical stimulus, arriving at the same rate, evokes faster reaction times (Figure 2) and larger MMN (Figure 3b) when it is embedded in a statistical pattern, which makes its occurrence less likely. Furthermore we demonstrated that sensitivity to statistical context, as indexed by different MMN amplitudes, goes beyond local adaptation of afferent activity in narrow frequency bands (Figure 5), although adaptation plays an important role. These observations are consistent with the idea that observers build an internal model of the predominant stochastic distribution of stimuli, and implicitly and automatically monitor the environment for stimuli that are outliers to this distribution.

Earlier behavioural studies [23], [41] have shown that a high tone embedded in a sequence of low tones (or, indeed, a similar regularity-violating stimulus within another modality) improves the detection of a simultaneously-presented visual target. In our experiment, the visual stimulus was obvious enough that it was very rarely missed. Instead, we noted a consistent decrease in reaction time when the target onset was simultaneous with an auditory outlier. We speculate that a reaction-time effect of this sort is unlikely to depend on relatively slow executive or voluntary attentive mechanisms that exploit a learnt association; but instead reflects a low-level multimodal integration driven by the rapidity of auditory processing [42]. In particular, this suggests that the processes by which a statistical model of the environment is formed, and exceptions detected, lie at an early stage of sensory processing and can act autonomously of controlled attentionally-demanding executive processing.

This view is supported by the finding that the same probe stimuli embedded in identical random-frequency backgrounds evoke an MMN-like response (Figure 3b). There is an extensive literature showing rapid, and presumably automatic, sensitivity to changes in stimuli and to violations of deterministic [16] or sequence-based rules [18]. We add to these findings by showing that observers also implicitly learn statistical structure, and can detect outliers from a random distribution. This is evident in the MMN-like MEG signal that has similar timing to the conventional MMN evoked by sequence violations. Again, we find that the underlying physiological processes are sensitive to the overall statistics of stimuli and to the likelihood of an event conditioned upon its temporal context [22]: identical odd probes generated larger MMN responses when embedded in a narrower random-frequency context.

Source reconstruction suggested that the statistical-context-sensitive responses arose in the parietal and temporal cortices [9], [27], [30]. Intriguingly, sensitivity to sound statistics was not strong in the auditory cortex, as observed in previous studies that used conventional MMN paradigms, but rather in a region posterior to the primary auditory cortex, which agrees with prior work on statistical learning [29], [43]. These temporal-parietal areas have also been associated with stimulus-driven bottom-up saliency [28] and attention orientation to unexpected events [44]. Some behavioural studies have suggested that such involuntary redirection of attention may interfere [13], [14] with goal-directed behaviour, whereas others have shown evidence pointing towards facilitation [23], [41].

An important question is whether the sensitivity we see reflects global statistics, integrated over a wide range of frequencies and long time periods, or whether it is partly due to local adaptation. In particular, it might be that known stimulus-specific adaptive phenomena in the auditory cortex [45] underlie the physiological and behavioural changes that we observe. Three lines of argument point towards more global processing. First, although most stimuli in the auditory stream had frequencies drawn from either the narrow or the broad contextual distribution, the odd probe stimuli themselves were presented at the same rate within both contexts. Furthermore, even in the broad context only about 1% of random contextual tones fell within a semitone-wide band around the odd probe frequency. Thus the pattern of 2000-Hz tones themselves differed negligibly between the two contexts (a very different situation to that found in studies of stimulus-specific adaptation [38], [45] where it is the probabilities of the different tones themselves that are varied), and the difference in both behavioural and physiological response effects must have been due to the distribution of contextual tones at other frequencies. Second, we looked at the impact that the recency of local-frequency stimulation had on the magnitude of the MMN. We certainly noted signs of local frequency-specific adaptation in the responses to odd probe tones occurring within the broad context: there, odd probes proceeded by longer periods in which all stimuli fell outside a frequency window (i.e., large 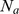) evoked a stronger response than those with recent in-window stimulation (Figure 5c, d). However, even when we looked only at odd probes in the broad context for which the preceding 15 or more tones (spanning 7.5 s) fell outside a window with rectangular bandwidth equivalent to a psychophysical auditory filter or wider, the size of the MMN was significantly larger than that evoked by odd probes in the narrow context. (Long time-scale adaptation has also been reported in the context of SSA [38], but may well point to more global processing even in that context). Third, we observed very little, if any, local adaptive difference in the magnitude of MMN evoked by odd probes in the narrow context. In this context most of the potentially adapting stimuli that fell within the frequency window of the analysis were themselves other odd probe tones. Thus, it seems that when presented in the narrow context, odd probe tones remain surprising even if another odd probe was presented very recently. Indeed, it seems possible that odd probes in the narrow context are heard as distinct, as though they were generated by a different process. Again, this finding points to a sensitivity to the global context [22], [31] within which specific stimuli are heard. Taken together, these results highlight the fact that the brain's ability to detect regular patterns in the environment, and register violations of these patterns, goes beyond repetitive [8] or sequence-based rules [16], [46]. Instead, the apparently implicit physiological process associated with the MMN seems to extract at least the first two moments of the distribution of stimuli in the environment, and then to drive stronger responses to tones that are more improbable within this encoded distribution.

Our results demonstrate the brain's ability to implicitly and efficiently encode the statistical regularity of events in the environment. In keeping with normative ideas of predictive coding [3], [5], [47] and of sensitivity to varying forms of uncertainty [6], we suggest that this computation might be essential for perceptual processing in a noisy environment, and for decision-making in an uncertain world.

## Methods

### Ethics statement

Informed consent was obtained from each subject, after full explanation of the experiment, according to the procedures approved by the University College London Research Ethics Committee.

### Participants

We recorded behavioural reaction time data from ten participants (5 females, 5 males, age range 24–32 years, and mean age 26 years), and MEG data from a separate pool of eighteen participants (8 males, 10 females, age range 19–47 years, and mean age 28 years). All participants were healthy volunteers, had normal to corrected vision and hearing, and were naive to the purpose of the study. Participants were monetarily compensated for their time.

### Experimental design

We designed a novel paradigm in which participants passively listened to a stream of pure tone sounds, whilst performing a visual change-detection task. The experimental design is depicted in Figure 1. The frequencies of most of the tone pulses were sampled from a Gaussian distribution in log-frequency, centred at 500 Hz and with one of two standard deviations: low (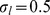 octaves) or high (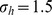 octaves). All tone pulses had an equal duration of 50 ms with smooth rise and fall periods of 10 ms each, were set to the same comfortable loudness level throughout the experiment, and were presented every 500 ms. Probe tones, whose frequencies were either equal to the mean of the distributions (standard probes: 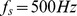) or two octaves above it (odd probes: 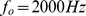), were embedded within the random-frequency streams. Both types of probe tone were inserted into the stream pseudo-randomly, with each presented 10% of the time. The probe tones were not distinguished from those of the background distribution, and thus participants experienced a slightly distorted Gaussian distribution of frequencies which combined two point-masses of 10% probability each (the standard and odd probes) with the Gaussian contributing 80% of the frequencies. Although this renders the overall distribution not strictly Gaussian, the 10% prevalence of probes was necessary to ensure a sufficient number of trials (and SNR) to see the MMN-like response. Only the width of the Gaussian part of the distribution differed between the two conditions. Behavioural and physiological comparisons were all based on responses to the probes alone, and these were identical in both conditions. Participants were told to ignore the sounds and respond only to the visual task, which required them to press a key each time they saw a brief change in the luminance of a fixation cross. The interval between successive luminance changes was randomly chosen between 2000 ms and 5000 ms, in steps of 500 ms. In the behavioural experiment (Experiment 1), these changes were coincident with the probe sounds, both standard and odd. In the MEG experiment (Experiment 2) luminance changes were made independent of the sound stream so as to avoid introducing confounding motor or attentional signals.

Experiment 1 lasted approximately 30 minutes and was divided into 4 blocks. Both high and low variance conditions were presented in all blocks for half of the time, and in randomised order. In Experiment 2, the high and low variance conditions were presented in two separate blocks that each lasted for 13 minutes (resulting in about 160 probe tones of each type per condition per participant). The order of these blocks was counter-balanced across participants.

The stimuli and task protocols were written in MATLAB, using the Cogent 2000 toolbox (http://www.vislab.ucl.ac.uk/cogent.php).

### MEG recordings and preprocessing

Measurements were acquired with a CTF 275-channel whole-head MEG system, with 274 functioning second-order axial gradiometers arranged in a helmet shaped array. Three energised electrical coils were attached to the fiducials (nasion, and left and right preauricular), in order to continuously monitor the position of each participant's head with respect to the MEG sensors. Auditory stimuli were binaurally presented at a comfortable loudness level through flexible tubing connected to piezo electric transducers positioned approximately 1 m below the sensor array. Data were collected at a sampling rate of 600 Hz, and recording epochs extracted stretching from 100 ms before to 350 ms after the onset of each sound (Figure 1, inset). For the spatio-temporal and source analyses, the data were filtered with a passband between 0.5 and 30 Hz, down-sampled to 200 Hz, and baseline-corrected with reference to the pre-tone interval (−100–0 ms).

### Spatio-temporal image conversion

The averaged sensor data, or ERFs, were converted into 3-dimensional spatio-temporal volumes. This was achieved by interpolating and dividing the scalp data per time point into a 2-dimensional spatial 64×64 matrix. We obtained one 2D image for every time bin. These images were then stacked according to their peristimulus temporal order resulting in a 3D spatio-temporal image volume with dimensions 64×64×91 per participant.

### Spatio-temporal statistical maps

For each subject, the 3D spatio-temporal image volumes were modelled with a mass univariate general linear model (GLM) as implemented in SPM [48]. We modelled the data with one regressor per condition: standard (s) and odd (o) probes, under high (h) and low (l) contextual variances, yielding coefficients 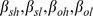, as well as two nuisance regressors that accounted for the block and variance factor confound. We then performed within-subject F-contrasts for (1) the main effect of surprise (regardless of contextual variance) [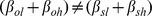], and (2) the interaction or differences between odds and standards in the context of a low, as compared to a high variance distribution, [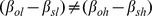]. We then carried these contrasts over to a one-sample between-subject F-test statistic and assessed the significance of the tests across the group. This approach allowed us to make inferences on all 3 data dimensions, i.e., 2-dimensional sensor space over the whole peristimulus time dimension. The same sort of statistical analysis was performed on the 3D spatial image volume obtained after the source localization step (see below). All sensor effects are reported at a threshold of p<0.05, with a family-wise error (FWE) correction for multiple-comparison for the whole volume.

### Source reconstruction

We obtained source estimates on the cortical mesh by reconstructing scalp activity with a single-shell head model, and inverting a forward model with multiple sparse priors (MSP) assumptions for the variance components [25] under group constraints [24]. This allowed for inferences about the most likely cortical regions that generated the data observed in the MMN time-window [100–200 ms], pre-selected according to predictions derived from previous MMN studies and our scalp results. We obtained images from these reconstructions for each of the four conditions in every subject. These images were smoothed at FWHM 8×8×8 mm^3^. We then computed the main effect of surprise and interaction (surprise by contextual variance) using conventional SPM analysis [48]. Similarly to the spatio-temporal statistical tests (described above), we were able to search for significant effects over the whole brain 3-dimensional space. Effects (t-statistics) are displayed at an uncorrected threshold of p<0.05 (Figure 4). These weaker significance criteria were used for post-hoc visualisation, once the effects had been established under robust criteria at the scalp level.

### Spatial filtering

The local adaptation analysis (described below) required the construction of ERFs based on relatively small numbers of responses. As single-channel ERFs for low trial counts exhibited substantial noise, we used a single-output spatial filter to combine all channels in a way that maximised the average signal-to-noise ratio across the four probe conditions [40]. We constructed two covariance matrices as follows. Let 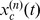 be a vector representing the multidimensional MEG measurements at sample 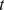 (of 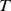 in the epoch), associated with the *n*th repetition (of *N_c_* for the condition), of probe tones under the condition labelled by *c* (either standard or odd probes, in either the high- or low-variance context); and let 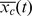 be the mean measurement over all *N_c_* stimuli of that condition. Then the condition-specific signal power matrix is
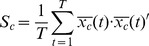
and the noise covariance matrix is
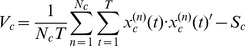
We constructed overall signal power *S* and noise covariance *V* matrices by averaging the four corresponding condition-specific matrices. The maximum signal-to-noise spatial filter was then the eigenvector *w* corresponding to the largest eigenvalue solution of the generalised eigenvalue problem:
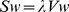
ERFs were constructed for each condition using the one-dimensional filtered signal (Figure 5c):
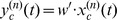
The filter *w* represents a compromise between spatial patterns of greater signal and those of lesser noise (Figure 5a). However, it is possible to recover the effective signal direction either by correlating the filtered output with the multidimensional MEG signal or (equivalently, and more simply) pre-multiplying *w* by the noise matrix *V* (Figure 5b).

### Local adaptation analysis

To examine the contribution of local adaptive mechanisms to our findings, we labelled responses to odd probe tones according to the number (*N_a_*) of preceding tones of all types that fell outside a window of full-width 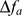 in log-frequency centred at the odd probe frequency (see Figure 1). We then averaged responses for which *N_a_* exceeded a threshold value (Figure 5c, d) to yield separate ERFs for each local adaptation condition. The (negative) peak value was computed for each separate response by averaging samples that fell within 30 ms of the minimum of the corresponding ERF curve. These peak values were compared between the broad and narrow context using a one-tailed sampled permutation test in which the context labels for each response were randomly permuted 2500 times, with the fraction of permutations for which the difference between broad and narrow context responses was greater than that observed for the true assignment providing the corresponding *p*-value. As we were interested in the hypothesis that the difference would be significant in all adaptation groups, we used an independent threshold of 0.05 for significance.

All the analyses were performed with SPM (http://www.fil.ion.ucl.ac.uk/spm/) and in-house MATLAB code.

## Supporting Information

Figure S1
**Adaptation effects for a range of windows.** Curves show ERF peaks for odd probes in narrow (*blue*) and broad (*red*) context grouped by different threshold value of *N_a_*, number of preceding tones falling outside a frequency window of width 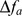, calculated for different frequency exclusion windows (*colour saturation*, see legend at top). Error bars show standard errors. Grey stars indicate pairs of ERFs that were significantly different at the p<0.05 level according to a random permutation test. Lines at the bottom show the number of probe tones (combined across all subjects) that contribute to each ERF.(TIFF)

Figure S2 **Evoked responses to standard and odd probes in the broad and narrow contexts.** Whole head 274-channel scalp responses (left). Right parietal channel (MRP57) shows averaged responses evoked by odd probes in the narrow (*green*) and broad (*turquoise*) contexts, and standard odd probes in the narrow (*blue*) and broad (*red*) contexts (right).
(TIF)
