# Supplementary figures and images for: Outlier Responses Reflect Sensitivity to Statistical Structure in the Human Brain
Source: PLoS Comput Biol. 2013 Mar 28;9(3):e1002999. doi: 10.1371/journal.pcbi.1002999 (PMC3610625; doi:10.1371/journal.pcbi.1002999)

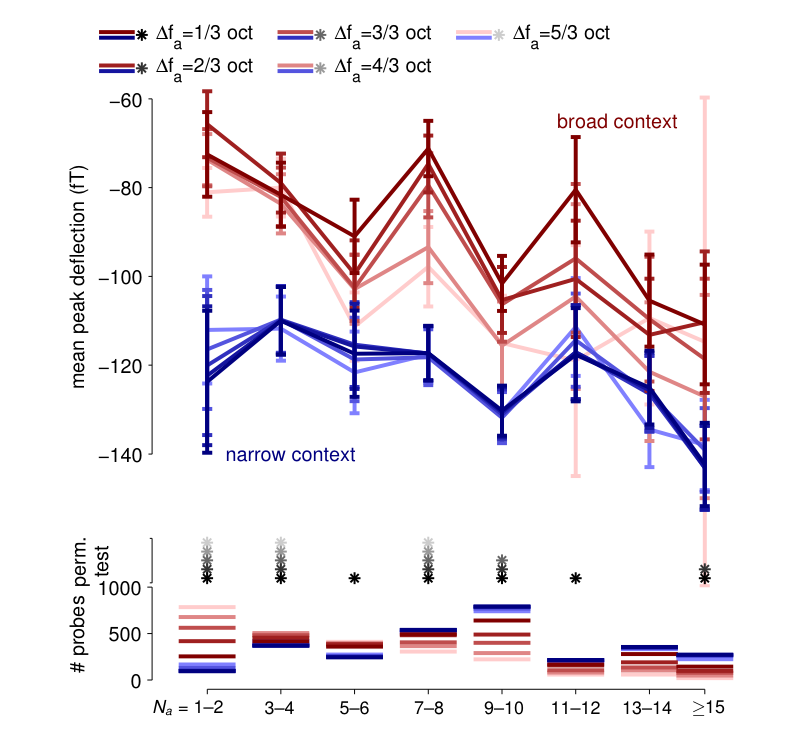

Supplement: Figure S1 — Adaptation effects for a range of windows. Curves show ERF peaks for odd probes in narrow (blue) and broad (red) context grouped by different threshold value of Na, number of preceding tones falling outside a frequency window of width , calculated for different frequency exclusion windows (colour saturation, see legend at top). Error bars show standard errors. Grey stars indicate pairs of ERFs that were significantly different at the p<0.05 level according to a random permutation test. Lines at the bottom show the number of probe tones (combined across all subjects) that contribute to each ERF. (TIFF) [file pcbi.1002999.s001.tiff]

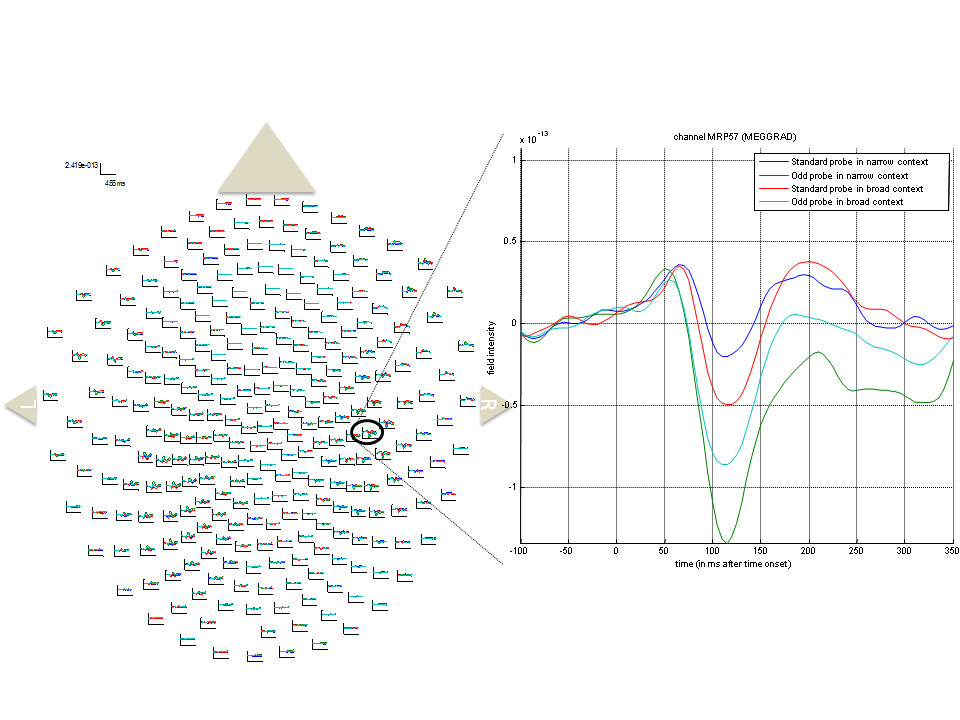

Supplement: Figure S2 — Evoked responses to standard and odd probes in the broad and narrow contexts. Whole head 274-channel scalp responses (left). Right parietal channel (MRP57) shows averaged responses evoked by odd probes in the narrow (green) and broad (turquoise) contexts, and standard odd probes in the narrow (blue) and broad (red) contexts (right). (TIF) [file pcbi.1002999.s002.tif]
